# Whole exome sequencing reveals the maintained polyclonal nature from primary to metastatic malignant peripheral nerve sheath tumor in two patients with NF1

**DOI:** 10.1093/noajnl/vdz026

**Published:** 2019-09-10

**Authors:** Abigail Godec, Reyka Jayasinghe, John S A Chrisinger, Bethany Prudner, Tyler Ball, Yuxi Wang, Divya Srihari, Madhurima Kaushal, Hilary Dietz, Xiaochun Zhang, Melike Pekmezci, Sonika Dahiya, Yu Tao, Jinqin Luo, Brian A Van Tine, Li Ding, David H Gutmann, Angela C Hirbe

**Affiliations:** 1 Division of Medical Oncology, Department of Medicine Washington University School of Medicine, St. Louis, Missouri; 2 Siteman Cancer Center, Washington University School of Medicine, Saint Louis, Missouri; 3 Department of Immunology and Pathology, Washington University School of Medicine, Saint Louis, Missouri; 4 McDonnell Genome Institute, Washington University School of Medicine, St. Louis, Missouri; 5 Department of Pathology, University of California San Francisco School of Medicine, San Francisco, California; 6 Cancer Center Biostatistics Shared Resource, Division of Public Health Sciences, Department of Surgery, Washington University School of Medicine, St. Louis, Missouri; 7 Department of Neurology, Washington University School of Medicine, St. Louis, Missouri; 8 Neurofibromatosis Center, Washington University School of Medicine, St. Louis, Missouri

**Keywords:** Genomics, MPNST, Metastasis, NF1, TRIM23

## Abstract

**Background:**

Malignant peripheral nerve sheath tumors (MPNSTs) are aggressive soft tissue sarcomas with high metastatic rates and poor overall patient survival. There are currently no effective therapies, underscoring the pressing need to define the molecular etiologies that underlie MPNST progression. The aim of this study was to examine clonal progression and identify the molecular events critical for MPNST spread.

**Methods:**

In two patients with temporally and spatially distinct metastatic lesions, we employed whole exome sequencing (WES) to elucidate the genetic events of clonal progression, thus identifying the molecular events critical for MPNST spread.

**Results:**

First, we demonstrated shared clonal origins for the metastatic lesions relative to the primary tumors, which were maintained throughout the course of MPNST progression, supporting the conclusion that cancer cells with metastatic potential already exist in the primary neoplasm. Second, we discovered TRIM23, a member of the Tripartite Motif family of proteins, as a regulator of MPNST lung metastatic spread in vivo.

**Conclusions:**

The ability to track the genomic evolution from primary to metastatic MPNST offers new insights into the sequence of genetic events required for tumor progression and has identified TRIM23 as a novel target for future study in this rare cancer.

**Key Points**

• Cancer cells contributing to metastasis and reduced survival are present early in disease evolution.

• TRIM23, a seminal member of the TRIM family, plays a role in enhancing metastatic behavior.

**Importance of the Study**

Malignant peripheral nerve sheath tumors (MPNSTs) are aggressive soft tissue sarcomas with a high metastatic potential. Despite aggressive therapy, the 5-year overall survival is dismal. As such, there is a pressing need to understand the genomic mechanisms that underlie metastatic progression in order to design rational therapies. We leveraged whole-exome sequencing of the tumors from two patients with sequential metastatic lesions to better define tumor clonality and to identify the molecular events critical for metastatic progression. This analysis led to the identification of point mutations and copy number alterations in *TRIM23* within the metastatic lesions. Additionally, we found that increased expression of nuclear TRIM23 correlated with worse overall survival in patients. Furthermore, *Trim23* knockdown in murine *NPcis* MPNST cell lines resulted in a decreased propensity for metastasis in vivo, establishing TRIM23 as a potential candidate for further study.

Neurofibromatosis type 1 (NF1) is a common cancer predisposition syndrome, affecting approximately 1 in 3,000 individuals worldwide.^1^ All individuals with NF1 are born with a germline mutation in one copy of their two *NF1* tumor suppressor genes, but develop benign tumors following somatic inactivation of the remaining *NF1* allele.^2^ Although benign tumors predominate in children with NF1, adults with NF1 are at risk for malignancy, most commonly malignant peripheral nerve sheath tumors (MPNSTs). These Schwann cell-derived neoplasms metastasize widely to the lung and bone, and the majority of patients die from metastatic disease within 5 years of initial diagnosis, even after surgery, radiation, and chemotherapy.

Given the elevated frequency of MPNSTs (10%–13% adults^3^) and their younger age of onset (~3rd decade of life^4,5^), NF1 has emerged as a tractable model system to define the molecular etiologies for MPNST pathogenesis, as these cancers most often arise from benign precursor lesions (plexiform neurofibromas) with biallelic *NF1* gene inactivation. Importantly, transformation to cancer requires additional genetic alterations involving other genes, such as *TP53, CDKN2A,* and *EED/SUZ12*.^6^ Although numerous laboratories have focused on understanding the molecular evolution of MPNST from their benign counterparts, very few studies have attempted to characterize the intratumoral heterogeneity as it relates to MPNST metastasis.^7,8^ In an effort to define the genomic alterations important for tumor progression and explore intratumoral heterogeneity, we employed next generation sequencing technology to analyze MPNSTs from two patients with NF1 who each developed temporally and spatially distinct metastases over a period of 2–3 years.

## Methods

### Study Approvals

Blood, tumor, and other tissues were obtained from individuals diagnosed with NF1 according to established criteria^9^ and treated at Washington University/St. Louis Children's Hospital NF Clinical Program (St. Louis, MO). All human tumor samples were collected under an approved IRB protocol (#201203042) at Washington University, and all patients were appropriately consented. The Institutional Animal Care and Use Committee of Washington University has reviewed and approved our protocol for experiments utilizing animals. Animals were treated in compliance with IACUC policies.

### Patient Sample Preparation and Sequencing

Whole-exome sequencing was performed on tumor and matched normal DNA. Tumor DNA samples were obtained from formalin-fixed paraffin-embedded (FFPE) blocks or frozen tissue (when available) obtained at surgical resection or biopsy. Normal paired DNA was isolated from peripheral blood obtained by phlebotomy. The original samples were submitted to Otogenetics Corporation (Atlanta, GA) for human exome capture and sequencing. Genomic DNA (gDNA) was extracted from blood or frozen tissue using Qiagen DNeasy Blood & Tissue kit (Qiagen Ca#69506). Briefly, gDNA was subjected to agarose gel and OD ratio tests to confirm the purity and concentration prior to Bioruptor (Diagenode, Inc., Denville, NJ) fragmentation. Fragmented gDNAs were tested for size distribution and concentration using an Agilent Tapestation 2200 and Nanodrop. Illumina libraries were made from qualified fragmented gDNA using SPRIworks HT Reagent Kit (Beckman Coulter, Inc. Indianapolis, IN, catalog# B06938) and the resulting libraries were subjected to exome enrichment using SureSelectXT Human All Exon version 5 (Agilent Technologies, Wilmington, DE, catalog# 5190-6210) following manufacturer's instructions. Enriched libraries were tested for enrichment by qPCR and for size distribution and concentration by an Agilent Bioanalyzer 2100. The samples were then sequenced on an Illumina HiSeq2500, which generated paired-end reads of 125 nucleotides (nt). Data were analyzed for data quality using FASTQC (Babraham Institute, Cambridge, United Kingdom).

### Alignment, Variant Calling, and Bioinformatics Analysis

Sequence reads were mapped to the human reference genome (GRCh37/hg19) using Novoalign tool v3.02.06 (http://www.novocraft.com/products/novoalign/) followed by marking and removing PCR duplicates by PICARD tools v2.4.1 (http://broadinstitute.github.io/picard/). Local realignment around insertions/deletions was achieved by Genome Analysis Toolkit (GATK) v3.6-0.^10^ Somatic single-nucleotide variants were identified using Mutect v1.1.4^11^ and detection of insertions and deletions used Indelocator (https://www.broadinstitute.org/cancer/cga/indelocator). The tools compared each individual primary tumor or metastatic sample to the matched germline sample. Variants were then annotated using wAnnovar (http://wannovar.wglab.org/)^12^ which uses information from publicly available databases including 1000 genome, ExAC, dbSNP, COSMIC, and ClinVar. Copy Number Variants were detected and plotted using CNVkit^13^ and recurrent copy number changes were identified using GISTIC.^14^ Clonality was addressed using SciClone.^15^ After identifying targets of interest, Kaplan Meier Curves were generated using GEPIA (http://gepia.cancer-pku.cn/) on the TCGA RNA-seq expression dataset for a 95% confidence interval.

### Immunohistochemical Evaluation

Hematoxylin and eosin–stained sections were retrieved and were reviewed by a bone and soft-tissue sarcoma pathologist (JC) to confirm the diagnosis. Additional formalin-fixed paraffin-embedded sections were obtained from the patient blocks at Washington University in St. Louis along with previously described tissue microarrays generated at UCSF.^4^ Immunohistochemical stain for TRIM23 (ThermoFisher, PA5-34624, 1:150, rabbit polyclonal) was performed with appropriate positive and negative controls. Slide reviewers were blinded to the patients' clinical status. Nuclear staining for TRIM23 was evaluated for staining intensity (0 = negative, 1 = weak, 2 = moderate, and 3 = strong) and percentage of positive tumor cell nuclei (0 = 0%, 1 = 1%–24%, 2 = 25%–49%, 3 = 50%–74%, and 4 = 75%–100%).^16^ For survival analysis, samples were classified as “low” if the staining intensity was 0 or 1. Likewise, samples were classified as high if the intensity was 2 or 3. Immunohistochemistry for histone 3 lysine trimethylation (H3K27 me3) was performed using rabbit monoclonal antibodies (C36B11, 1:50 dilution; Cell Signaling Technology, Danvers, Mass) and Trisethylenediaminetetraacetic acid (EDTA) antigen retrieval. Tumors with nuclear staining in <5% of the tumor cells, in the presence of an internal positive control (e.g., endothelial cells), were scored as having a loss of H3K27me3. Immunohistochemical staining for Cleaved-caspase 3 at 1:500 (Cell Signaling Technology, Danvers, MA) with citrate antigen retrieval or prediluted Ki67 (790–4286; Ventana) with CC1 buffer (950- 124, Ventana) antigen retrieval was performed on subcutaneous tumor sections and the number of cells in five high power fields were counted by three blinded reviewers.

### Cell Culture, Transduction, and Immunoblot Analysis

Murine MPNST tumor lines established from C57BL6/J *Nf1+/−;Trp53+/− (NPcis)* expressing GFP-Luciferase^17^ were used for the in vitro, and in vivo MPNST tumor growth experiments. Cells were transduced with either lentiviral control shLacZ (Genome Institute at Washington University) or separate lentiviral shRNAs targeting *Trim23* (Sigma; TRCN0000308323, TRCN0000308321, TRCN0000308320). The cell line exhibiting the best knockdown, as determined by Western blot and mRNA analysis, was selected and expanded for subsequent experiments (Sigma TRCN0000308323). No mycoplasma contamination was detected in any cell lines and cells were used within 3–7 passages from thawing. Western blots were performed from lysed cell pellets using 1%NP-40 lysis buffer supplemented with protease inhibitors. Protein separation and detection were performed using an automated capillary electrophoresis system (Simple Western system and Compass software; ProteinSimple). Wes Separation Capillary Cartridges for 12–230 kDa were used for detection and internal Total Protein Detection were used for quantitation controls according to manufacturer instructions. TRIM23 antibody (dilution 1:50) was used for signal detection along with an HRP-conjugated secondary anti-rabbit antibody, and was visualized using ProteinSimple Compass software.

### RNA Extraction and Quantitative RT–PCR

Total RNA was extracted from *NPcis* cells infected with shLacZ or shTrim23 using TRIzol according to manufacturer's instructions (ThermoFisher Cat. #15596026). Reverse transcription reactions were performed with Superscript III (Invitrogen) according to manufacturer's protocol. Real-Time PCR reactions were executed using CFX96 Real-Time PCR Detection System (BioRad) and detected using PowerUp SYBR Green (Applied Biosystems; 100029284). The following primers were used:

*Gapdh* forward, 5′-ATGACATCAAGAAGGTGGTG-3′;
*Gapdh* reverse, 5′-CATACCAGGAAATGAGCTTG-3′;
*Trim23* forward, 5′-ATACAGGTTCTGGAGTGTGG-3′;
*Trim23* reverse, 5′-CCTGAGTCTCCTAGGTCTGTTAC-3′

Analysis was performed on Bio-Rad CFX Manager Software (BioRad 2017).

### In Vitro Assays

Cells were counted using the Countess Automated Cell Counter (Invitrogen), plated in 96-well plates (~ 15,000 cells/well), and allowed to grow overnight. For proliferation assays, cells were plated in phenol-red free DMEM with 10% FBS. Cells were imaged every hour for 48 hours using the Incucyte FLR imaging system (Essen Bioscience, Ann Arbor, MI) and analyzed for quantitation using Incucyte ZOOM Analysis Software (Essen Biosciences). Phase images were used to determine percent confluence and subsequent wells were normalized to initial confluence. For clonogenic assays, control (shLacZ), and shTRIM23 Cell lines (1,2,3) were seeded at 200 cells/well in a 6 well dish (triplicates) in 3-mL DMEM medium. An initial image was taken 24 hours after plating (0 h) and allowed to grow for 2 weeks where a final image was taken (2 wk) using the IncuCyte system (Sartorius, Ann Arbor, MI). For quantification of colonies, using the image analysis software provided with the Incucyte system, cell masks were calculated for each respective well to quantify confluency at each time point.

### Immunofluorescence

Cells were plated at low density on chamber slides (MilliporeSigma) and fixed with 4% paraformaldehyde, permeablized for 5 min at 4°C with 0.1% Triton-X100. Cells were stained with previously reported TRIM23 antibody (1:150; 4°C overnight) and β-actin (Santa Cruz; sc-47778; 1:500; 37°C 45min) and fixed with ProLong Gold Antifade Mountant with DAPI (ThermoFisher; P36930).

### In Vivo Studies

Subcutaneous tumors were performed by injecting 1 × 10^5^ cells into the medial flank 4-week-old male *B6N-Tyrc-Brd/BrdCrCrl* mice (Charles Rivers)^17^ using JW23.3-shLacZ and JW23.3-sh*Trim23*-3405 tumor cells (*n* = 5 mice/group) imaged at days 4, 8, 14, 21, and 28. The metastatic model involved injecting 1 × 10^5^ cells intraventricularly into 3-week-old anesthetized male *B6N-Tyrc-Brd/BrdCrCrl* mice for imaging at days 2, 5, 9, 12, and 16. Each experiment consisted of 5 mice per group and each experiment was repeated to validate the results. Luciferase signal was measured in photon flux and was quantitated using automatic and constant ROIs for subcutaneous and intraventricular models respectively.

### Statistics

Kaplan–Meier survival curves based on TRIM23 patient immunohistochemistry was analyzed using GraphPad Prism Version 7.03. The association between TRIM23 protein expression and H3K27 me3 status was assessed by chi-square test. Kaplan–Meier survival curves were generated for overall survival by H3K27 me3 protein expression groups in MPNST. Patients were censored by date of death (Social Security Death Index, expiration note in chart or obituary) or date of last follow-up. Overall survival is defined as date from diagnosis to date of death by any cause. The survival difference between groups was compared using the log-rank test. Statistical analyses were performed with GraphPad Prism Version 7.03 and SAS (version 9.4; SAS Institute, Cary, NC). Statistical analyses for qRT–PCR analyses were performed using GraphPad Prism. Bar graphs are presented as the mean +/− SEM, and the Students *t*-test was used for statistical significance. Statistical significance was determined for all Incucyte assays and all in vivo experiments using one-way and two-way ANOVA analysis, respectively, on GraphPad Prism.

## Results and Discussion

### Clonal Origins of MPNST

**Figure 1.**
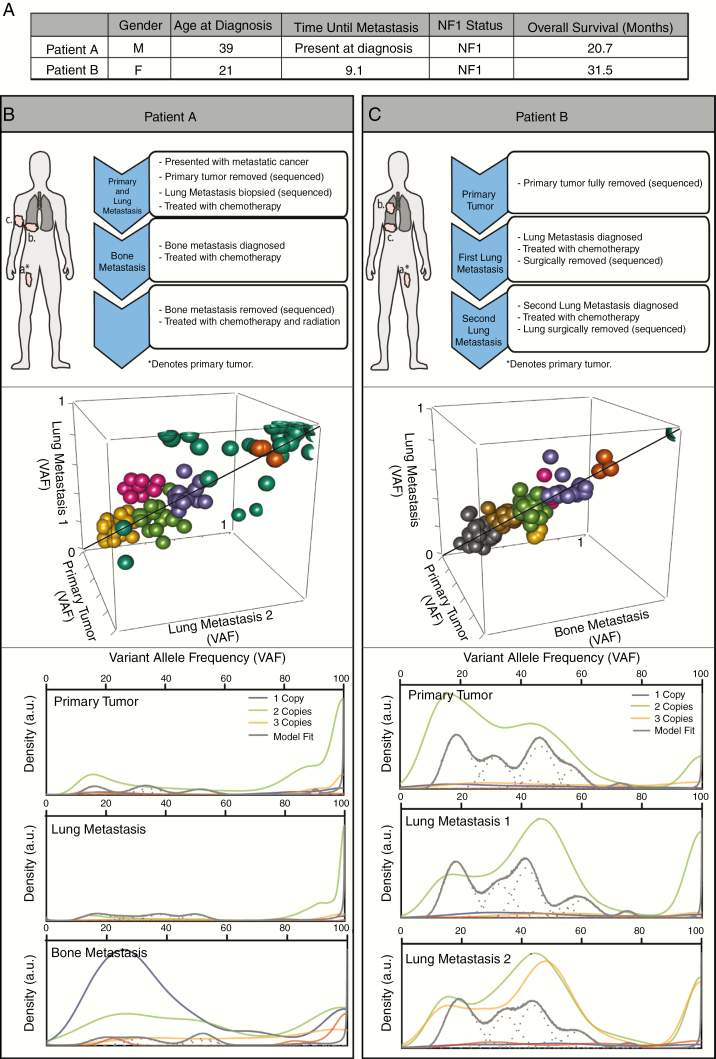
Patient characteristics and establishing clonal origin of metastases within MPNSTs. (A) Patient characteristics at time of diagnosis and overall disease progression. (B, C Upper) MPNST Treatment progression for patients A and B after diagnosis. Three spatially distinct tumor samples were aligned to normal blood control from each individual. (B, C Middle) Still-frame of a 3-dimensional plot of VAF clusters of genomic signatures in copy number neutral regions compared across samples within a patient. (B,C Bottom) Kernel density plots of proportion of mutations at each VAF across genome. Densities were summated over all clusters for each cluster/component. Line color based on predicted ploidy (posterior predictive densities) of the cell: one (dark grey), two (green), or three (yellow).

WES was performed on resection and biopsy materials from the primary tumor and associated metastatic lesions from two patients with NF1, established using NIH consensus diagnostic criteria^18^ (Figure 1A). Samples from patient A included a right thigh primary MPNST at age 38, as well as a right lower lobe lung metastasis at age 38 and a right humeral bone metastasis at age 39, whereas those from patient B included a left thigh primary MPNST at age 20, a right upper lobe lung metastasis at age 21 and a right lower lobe lung metastasis at age 22 (Figure 1B and C, upper panels; Supplementary Figure 1 [See online supplementary material for a color version of this figure.]). *NF1* mutation status and the presence of co-existing mutations associated with malignant transformation, including copy number loss at the *CDKN2A* locus, were demonstrated in both MPNSTs (Supplementary Figure 2 [See online supplementary material for a color version of this figure.]).

To investigate MPNST clonality, samples were clustered based on variant allele frequencies (VAF) within each tumor using SciClone, and compared against the other tumor samples within the same patient (Figure 1B and C, middle panels). Unexpectedly, we saw no emergence of a dominant clone enriched within the metastatic lesions. Rather, all of the clusters fell close to the identity line, suggesting similarity in the genomic mutation distribution within each patient. Next, the density of VAFs across the genome was examined for each tumor (Figure 1B and C, bottom panels). Genes mutated at similar rates show greater density on the plot. In patient A, mutational densities were grouped at high VAF counts in both the primary and lung metastasis, again supporting a shared genomic mutational profile. Read counts within the bone metastasis were unusually low, likely due to artifact from the decalcification process, thus precluding further analysis. However, in patient B, the primary tumor contained a subset of genes with VAFs between 20% and 60% and a smaller set of genes with a VAF between 85% and 100%. Between the primary tumor and the metastases, there was a right shift in density, suggesting enrichment of some clones. Nonetheless, the overall similarity of the distribution implied a shared genomic profile as the cancer evolves from primary tumor to metastatic lesion. These data argue that the primary tumor is polyclonal, and the subsequent metastatic lesions share the clones already present in the primary tumor. This observation is consistent with other recent reports, which demonstrate polyclonal metastases.^19,20^ Given the genomic instability present in these rapidly dividing cancer cells, this clonality suggests more similarities among the samples than would have been predicted. Taken together, it is likely that the cancer cells that drive MPNST metastasis exist in the primary tumor, supporting the notion that cancer progression may be targetable early during disease evolution. Moreover, the variation of clonal ratios in each MPNST sample may explain the differential responses of metastatic lesions to chemotherapy, suggesting that therapies for these deadly cancers may require a combination of distinct anti-neoplastic agents.

### Genomic Analysis Identifies TRIM Family Members as Potential Drivers of Metastasis

**Figure 2.**
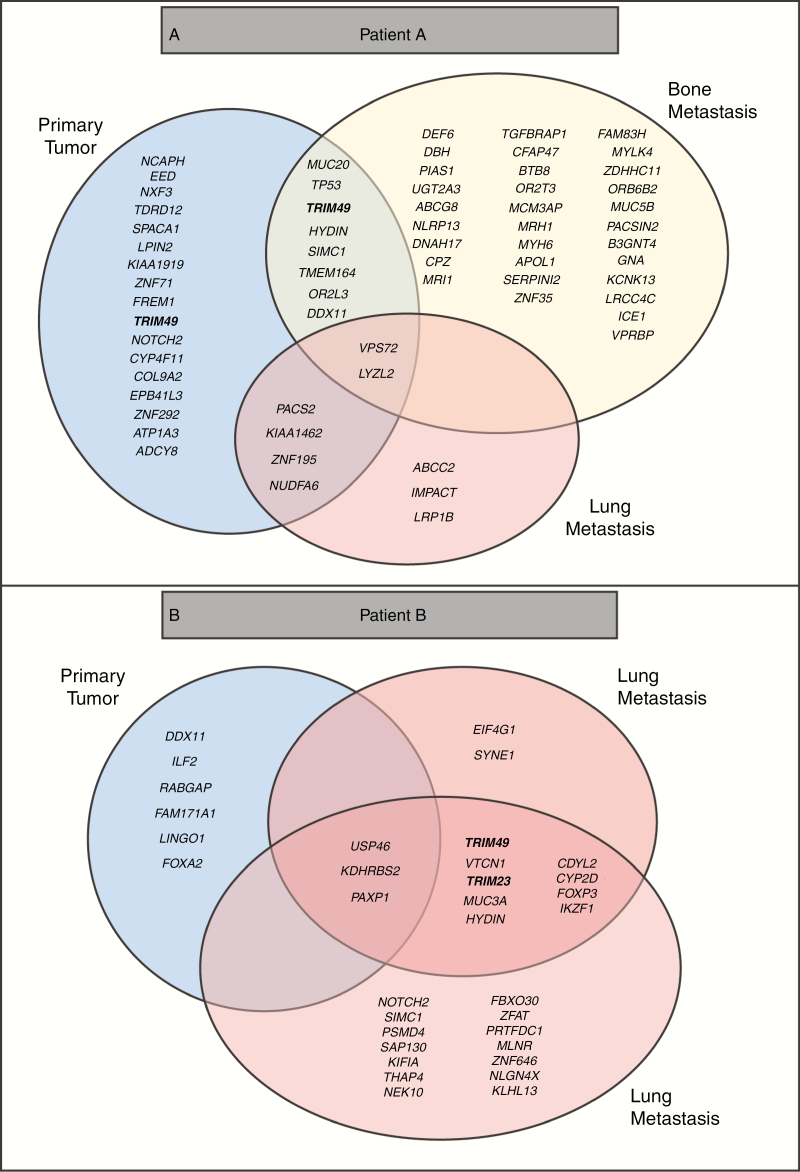
Genomic mutations over the course of metastatic MPNST. (A, B) Nonsynonymous exonic somatic called variants in each sample. Variants in the primary tumors are depicted in the blue circles. Mutations in the bone metastasis are depicted in the yellow circle. Mutations in the lung metastases are depicted in the red circles. TRIM genes, including *TRIM23*, mutated in the samples have been bolded.

**Figure 3.**
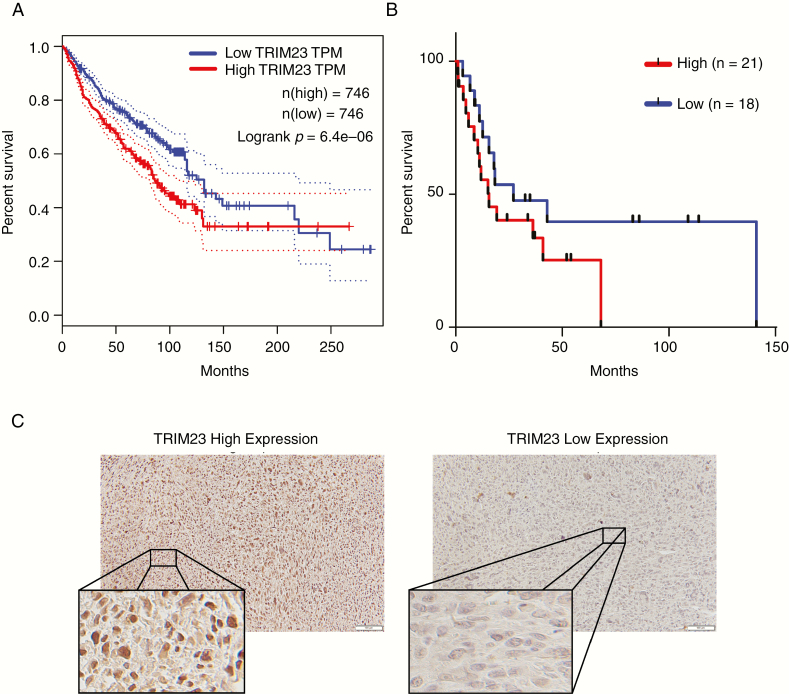
Identification of TRIM Family as potential drivers of progression and survival. (A) Kaplan–Meier survival curves for TRIM23 expression in sarcoma (SARC), glioblastoma (GBM), and breast carcinoma (BRCA) from TCGA GEPIA database. (B) Kaplan–Meier survival curves for TRIM23 expression from a cohort staining analysis of MPNST patients from Washington University in St. Louis and UCSF (*P* = .15; *n* = 39). (C) Representative examples of TRIM23 staining in MPNST.

To understand the importance of MPNST clonal diversity, nonsynonymous exonic SNP mutations were examined in both patient tumors. Point mutations were filtered by known polymorphisms, based on prevalence in the population using 1000 genomes and COSMic. Only mutations with a VAF > 0.03 were grouped within each sample and then overlapped for each patient (Figure 2A and B). Using these stringent criteria, no point mutations were shared across all samples. However, mutations in the TRIM family of proteins, a class of E3-Ubiquitin ligases, were present at higher rates in all of the metastatic samples. TRIM proteins are ubiquitously expressed in normal tissue, where they have cell type- and context-dependent functions. However, relevant to MPNST, TRIM proteins have been implicated as both positive and negative regulators of cancer progression.^21–23^ In light of recent reports demonstrating ubiquitination in cancer progression and metastasis,^24^ we pursued copy number alterations in the TRIM family members in all of the samples. Gains were called when log_2_ > 1.0 and losses were called when log_2_ < −1.0. Of the 61 TRIM family members, 14 members showed significant copy number alterations across samples (Supplementary Figure 3 [See online supplementary material for a color version of this figure.]). *TRIM23* is particularly compelling, as it is the only member of the family with GTP binding activity,^22^ and was the only TRIM family member identified in both point mutation and copy number analysis. Next, we queried a set of high-grade malignancies, which occur at an increased frequency in patients with NF1, including glioblastoma (GBM), breast cancer, and other sarcomas (SARC). Using the cancer genome atlas (TCGA) database available through the GEPIA portal, we analyzed the correlation between TRIM23 expression and overall survival^25^ and found that TRIM23 expression was associated with worse overall survival in this combined data set (Figure 3A, *P* = .0000064). Next, we examined TRIM23 expression in forty MPNSTs from Washington University and UCSF, and employed Kaplan–Meier analysis to define the relationship between TRIM23 expression and overall survival. Although patients with higher TRIM23 expression trended toward worse overall survival (*P* = .15), no statistically significant difference was observed in this small data set (Figure 3B and C). A larger multi-institutional study will be required to establish its prognostic utility in this rare cancer. The polycomb repressive complex 2 (PRC2)/polycomb repressive complex 2 subunit (SUZ12) has recently been shown to play an important role in MPNST pathogenesis.^26,27^ Given that PRC2 loss occurs in 60%–70% of MPNSTs and affects transcriptional regulation, we sought to determine whether there was a correlation between PRC2 loss and TRIM23 expression, which could suggest a link between these events. As a surrogate for PRC2/SUZ12 loss, we performed immunohistochemistry to examine H3K27 me3, a known downstream target of SUZ12. There was no correlation between these events in this cohort as assessed using a chi-square test (Table 1; *P* = .4754). Additionally, there was no association between H3K27 me3 status and overall survival (Supplementary Figure 4 [See online supplementary material for a color version of this figure.])

**Table 1.** TRIM23 expression in H3K27 staining

TRIM23 Expression Intensity	H3K27 Staining		
	Loss	High	Total
Low	13	8	21
	61.9%	38.1%	
High	14	7	21
	66.67%	33.33%	
Total	27	15	42

### Trim23 Knockdown Reduces MPNST Lung Metastasis In Vivo

**Figure 4.**
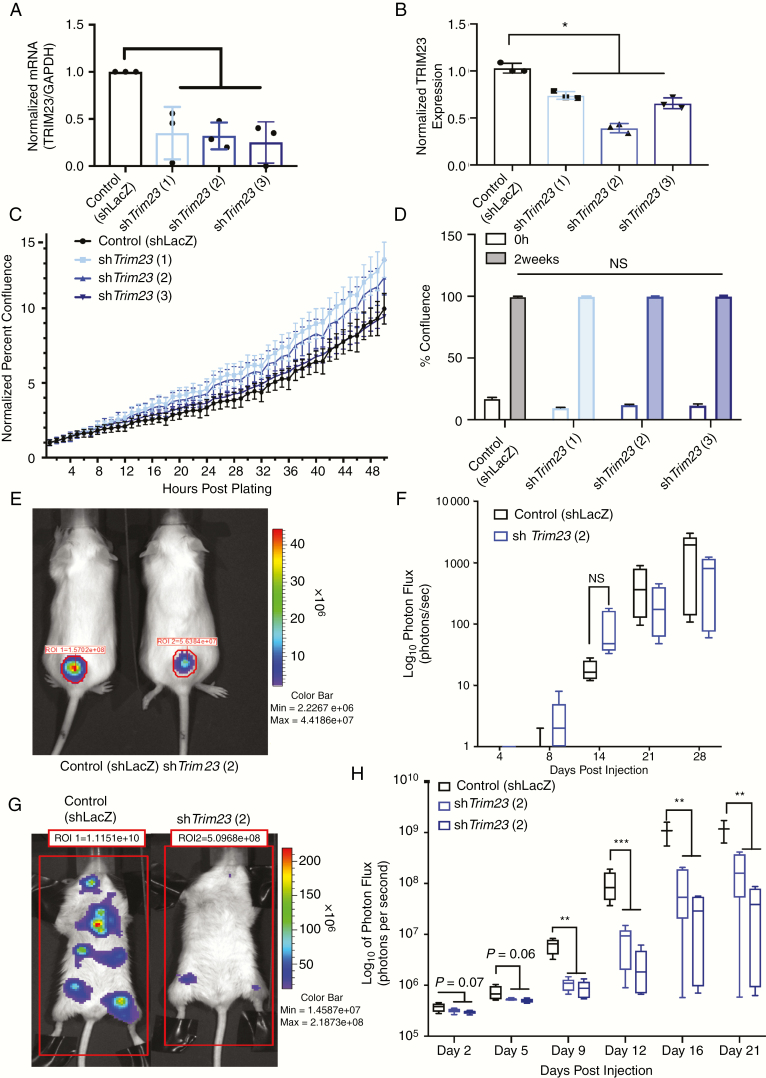
Loss of Trim23 leads to intrinsic tumor biology changes. (A) Relative mRNA expression of *Trim23* in *Nf1/Tp53*-mutant *NPcis* murine MPNST cell lines transduced with sh*LacZ* control or 3-unpooled sh*Trim23* viral particles. (B) Capillary Western blot of aforementioned cells, blotting for Trim23 with quantification. (C) An Incucyte cell proliferation assay measuring cell confluence over time. (D) Colony formation assay quantifying percent confluence. (E) Representative images of subcutaneous injected mice tumor burden at 16 days postinjection. (F) Quantification of BLI (photon flux) for subcutaneous injection models (*n* = 5 per group). (G) Representative images of left ventricle injected mice tumor burden at 16 days postinjection (*n* = 5 per group). (H) Quantification of BLI (photon flux) for left ventricle injection models (*n* = 5, * *P* < .05, ** *P* < .001, *** *P* < .0001).

To assess the potential functional significance of *Trim23* expression in MPNST pathogenesis, we first evaluated protein expression in three murine *Nf1/Tp53-mutant NPcis* MPNST cell lines engineered to express luciferase.^17^ All three lines had varying levels of *Trim23* expression (Supplementary Figure 5 [See online supplementary material for a color version of this figure.]). We specifically chose the line with the highest *Trim23* expression for subsequent experiments (Supplementary Figure 5A and B [See online supplementary material for a color version of this figure.]). This line is also the most aggressive of the three lines (Supplementary Figure 5C–F [See online supplementary material for a color version of this figure.]). Three shRNA constructs were used to knockdown Trim23 expression (Figure 4A and B; Supplementary Figure 6A [See online supplementary material for a color version of this figure.]) and utilized for in vitro experiments. The line with the most robust knockdown was chosen for in vivo testing. There was no change in MPNST cell proliferation following *Trim23* knockdown in vitro (Figure 4C and D; Supplementary Figure 6B and C [See online supplementary material for a color version of this figure.]) and no difference in tumor growth as subcutaneous explants in vivo (*P* = .17) (Figure 4E and F). Furthermore, there was no difference in levels of Ki67 or Cleaved Caspase 3 between the groups (Supplemental Figure 7 [See online supplementary material for a color version of this figure.]). In striking contrast, *Trim23* knockdown reduced tumor seeding (metastasis) following intraventricular injection (Figure 4G and H), supporting its role in tumor metastasis, rather than primary tumor expansion. This result was verified with two different *Trim23* knockdown lines.

Taken together, the proof of concept experiments described in this report make two important points. First, we establish that the cancer cells most likely to contribute to MPNST metastasis and reduced patient survival are already present in the primary tumor early during disease evolution. Second, we demonstrate that MPNST metastatic behavior is regulated by TRIM23, a seminal member of the TRIM family. Future studies are ongoing to define the underlying mechanism of action as an initial step towards targeting this clinically relevant population of cancer cells and developing biomarkers for disease progression.

## Funding

American Cancer Society (IRG-15-170-58 [to A.C.H.]), Francis Collins Scholar award through NTAP (3070–37791 [to A.C.H.]), the St. Louis Men's Group Against Cancer, and donations from the Brown and Wascher families. Partial funding from National Institutes of Health (P50 CA094056 to Molecular Imaging Center) and National Cancer Institute (P30 CA091842 to Siteman Cancer Center Small Animal Cancer Imaging [shared resource]). D.H.G. acknowledges generous support from the Diemer Family Fund.

## Supplementary Material

vdz026_suppl_Supplementary_Figure_Legends

vdz026_suppl_Supplementary_Figure_1

vdz026_suppl_Supplementary_Figure_2

vdz026_suppl_Supplementary_Figure_3

vdz026_suppl_Supplementary_Figure_4

vdz026_suppl_Supplementary_Figure_5

vdz026_suppl_Supplementary_Figure_6

vdz026_suppl_Supplementary_Figure_7
