# Supplementary material for: Whole exome sequencing reveals the maintained polyclonal nature from primary to metastatic malignant peripheral nerve sheath tumor in two patients with NF1
Source: Neurooncol Adv. 2019 Sep 10;2(Suppl 1):i75–84. doi: 10.1093/noajnl/vdz026 (PMC7317063; doi:10.1093/noajnl/vdz026)
Supplement: vdz026_suppl_Supplementary_Figure_Legends [file vdz026_suppl_supplementary_figure_legends.docx]

**Supplemental Figure 1. Representative images of patient tumors**

(A) Patient A H&E histology with 20X magnification and 40X magnification insert (100-micron scale bars). (B) Patient B H&E histology with 20X magnification and 40X magnification insert. (100-micron scale bars).

**Supplemental Figure 2. Genomic mutations characterizing primary and metastatic MPNST**

(A) Copy number confirmation of loss of the *CDKN2A* cooperating mutation. The *CDKN2A* locus is highlighted across all of the samples in yellow. (B) The variant allele frequencies of the non-synonymous exonic somatic called variants in each sample. Variants in the primary tumors are depicted in the blue columns. Mutations in the bone metastasis are depicted in the yellow column. Mutations in the lung metastases are depicted in the red columns. Italicized genes have mutations shared between all 3 samples within a patient. Bold type genes have mutations shared between 2 samples within a patient.

**Supplemental Figure 3. Copy Number Alterations in TRIM Family across samples**

(A) Copy number changes among all TRIM family members called with CNVkit. Gains were called when log2>1.0 and losses were called when log2 <-1.0.

**Supplemental Figure 4. Lack of correlation between H3K27me3 status in MPNSTs and overall survival**

(A) Kaplan-Meier survival curves for H3K27me3 expression from a cohort staining analysis of MPNST patients from Washington University in St. Louis and UCSF. (p=0.51; n=39)

**Supplemental Figure 5. Trim23 expression in murine MPNST cells correlates with metastatic potential**

(A) Immunofluorescence of Trim23 in JW12.1, JW15.3, and JW23.3 murine MPNST tumor lines established from C57BL6/J *Nf1+/-;Trp53+/- (NPcis)* mice is depicted in green with DAPI counterstain. (B) mRNA expression of Trim23 was determined by qPCR (Relative Trim23/GAPDH). (C-D) Quantification of tumor burden based on average fluorescence in subcutaneous and left-ventricle injection models for all three cell lines. (n=5 mice per group) (E-F) Representative images of tumor burden at day 16 post injection in all three cell lines in both tumor injection models.

**Supplemental Figure 6. Trim23 knockdown in murine MPNST does not affect cell proliferation *in vitro.***

(A) Trim23 is depicted in green, beta-actin is depicted in red, and DAPI is depicted in blue. (B) Confluency of murine MPNST cells after 48 hrs. Percent cell confluency was determined from orange confluency mask divided by total area. (C) Colony assay formation at initial seeding and 2 weeks after. Confluence mask shown in purple.

**Supplemental Figure 7. Trim23 knockdown in murine MPNST does not affect cell proliferation or cell survival in a subcutaneous model *in vivo.***

(A) Representative images of Ki67 staining from subcutaneous tumors formed after injection of JW23.3 MPNST cells with and without Trim23 knockdown. (B) Quantification of numbers of Ki67 positive cells across conditions. (C) Representative images of Cleaved Caspase 3 staining from subcutaneous tumors formed after injection of JW23.3 MPNST cells with and without Trim23 knockdown. (D) Quantification of numbers of Cleaved Caspase 3 positive cells across conditions. (E) Trim23 protein expression levels from subcutaneous tumors formed from injection of JW23.3 MPNST.
