# Supplementary figures and images for: Whole exome sequencing reveals the maintained polyclonal nature from primary to metastatic malignant peripheral nerve sheath tumor in two patients with NF1
Source: Neurooncol Adv. 2019 Sep 10;2(Suppl 1):i75–84. doi: 10.1093/noajnl/vdz026 (PMC7317063; doi:10.1093/noajnl/vdz026)

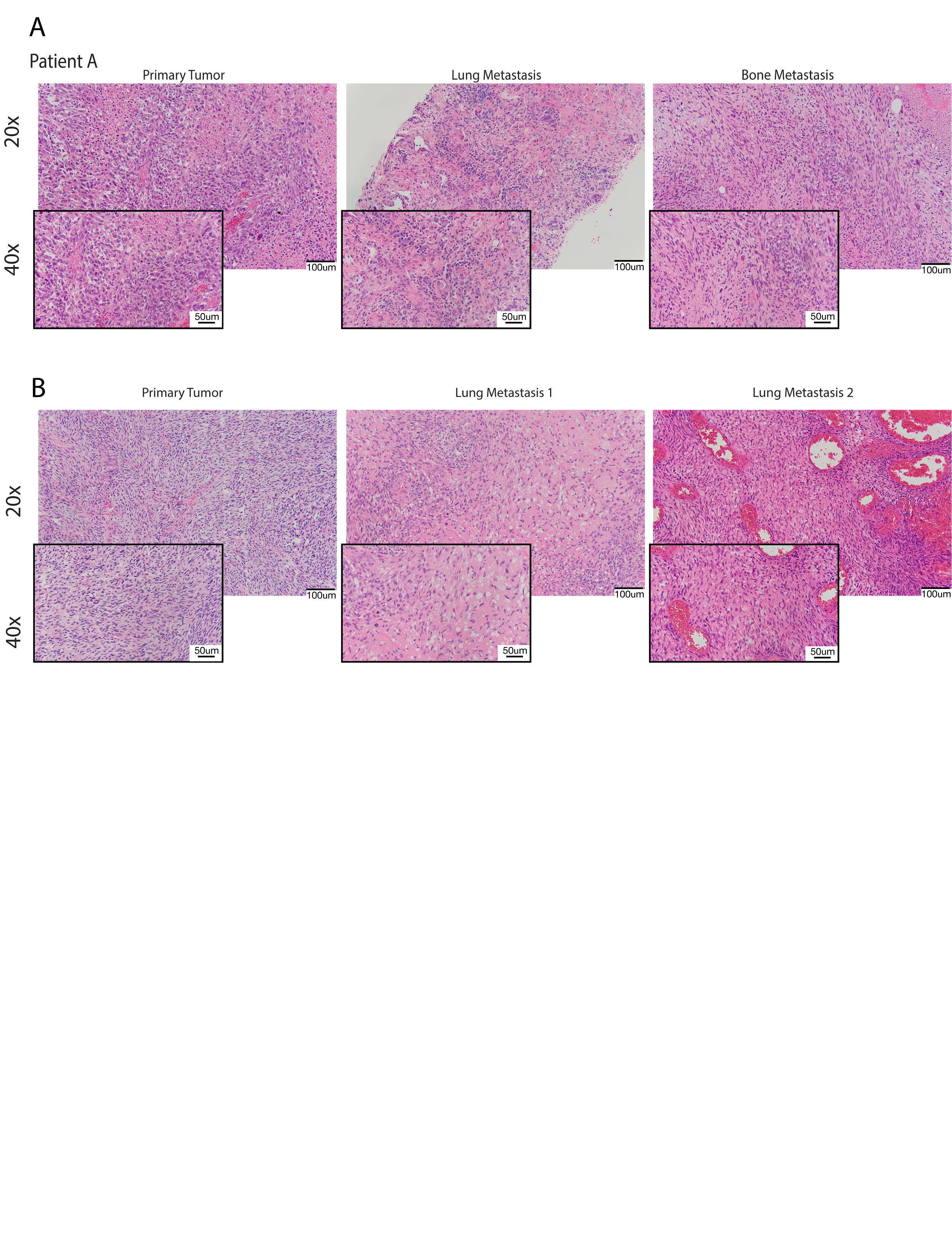

Supplement: vdz026_suppl_Supplementary_Figure_1 [file vdz026_suppl_supplementary_figure_1.png]

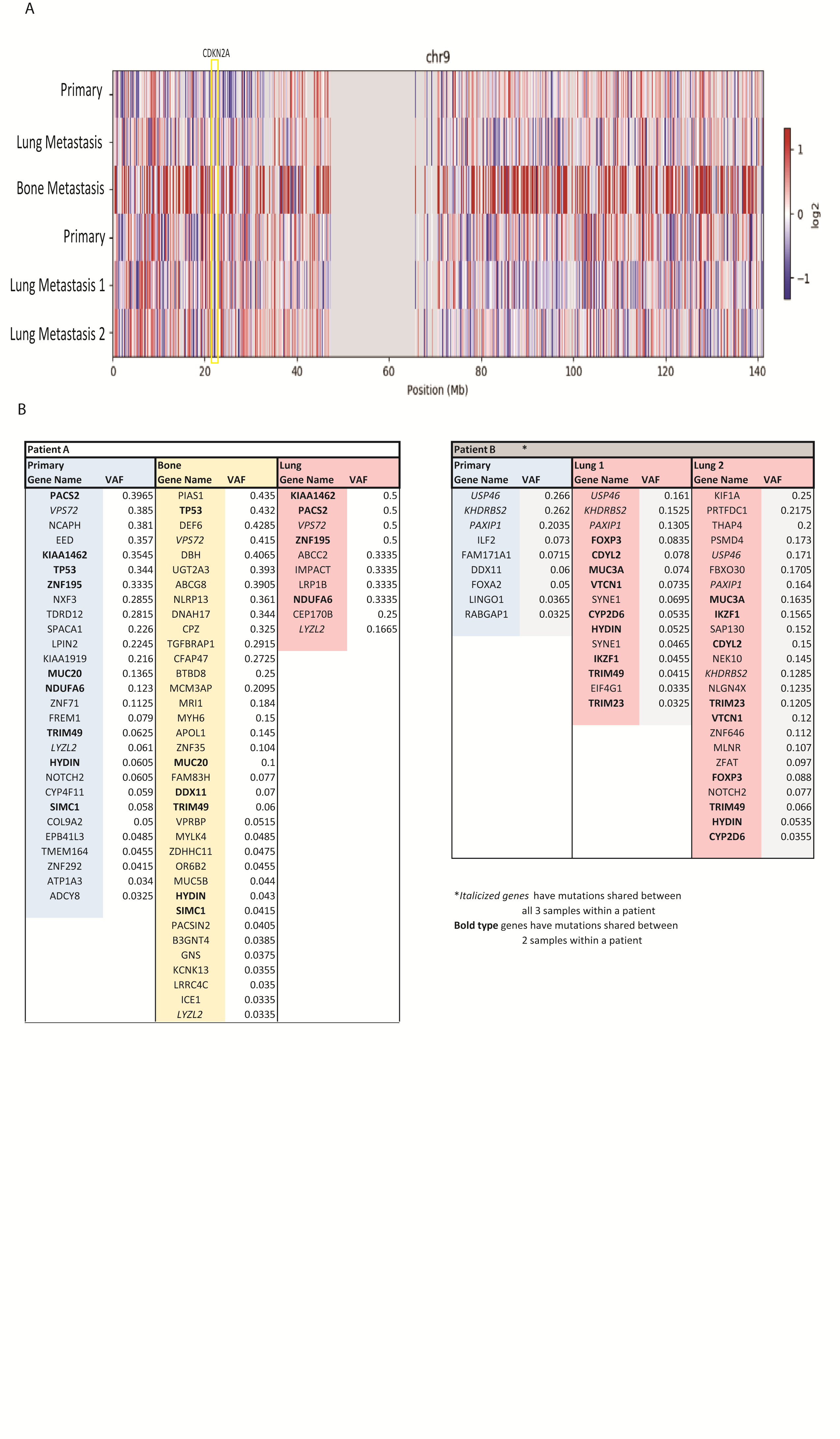

Supplement: vdz026_suppl_Supplementary_Figure_2 [file vdz026_suppl_supplementary_figure_2.png]

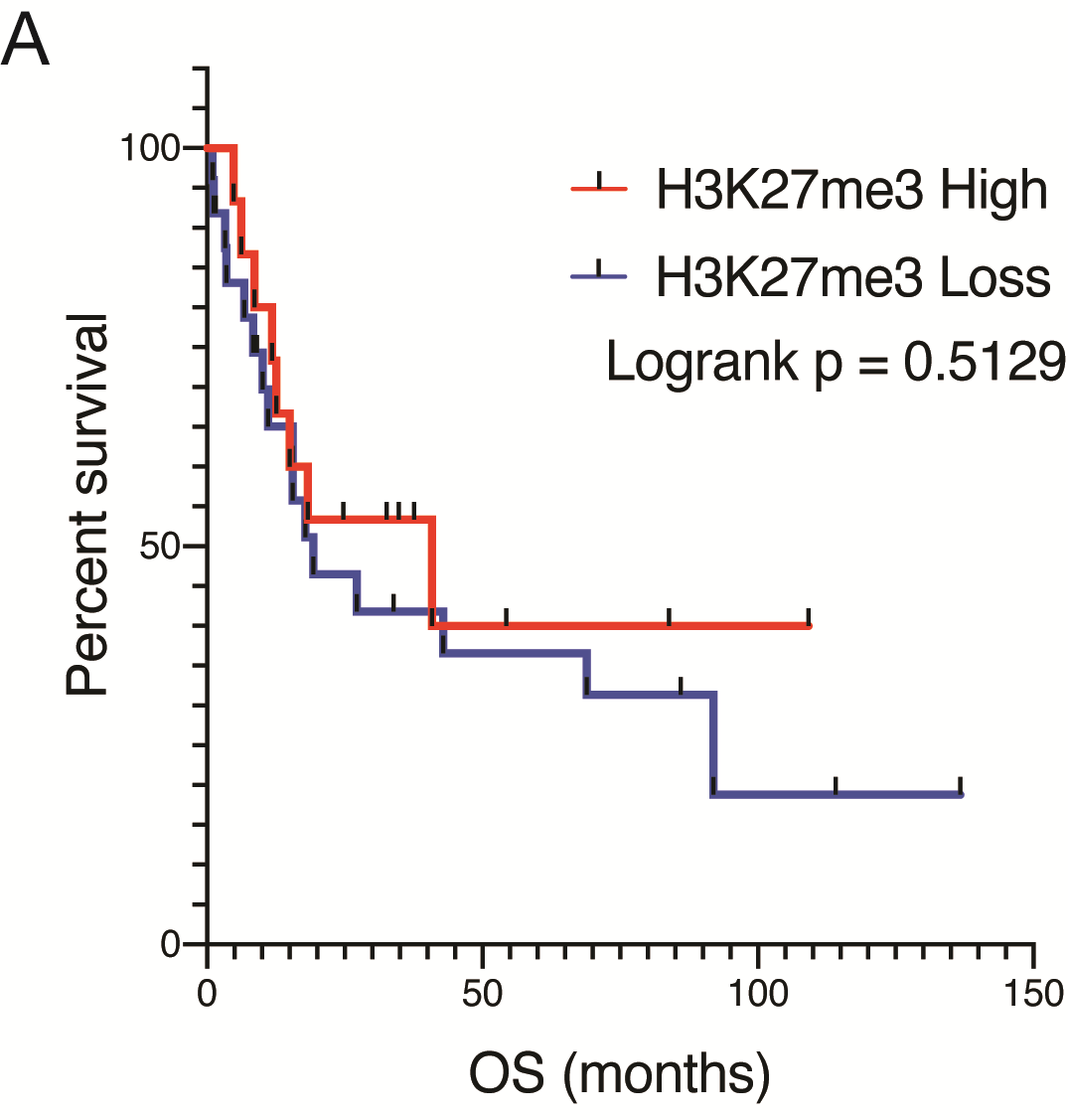

Supplement: vdz026_suppl_Supplementary_Figure_4 [file vdz026_suppl_supplementary_figure_4.png]

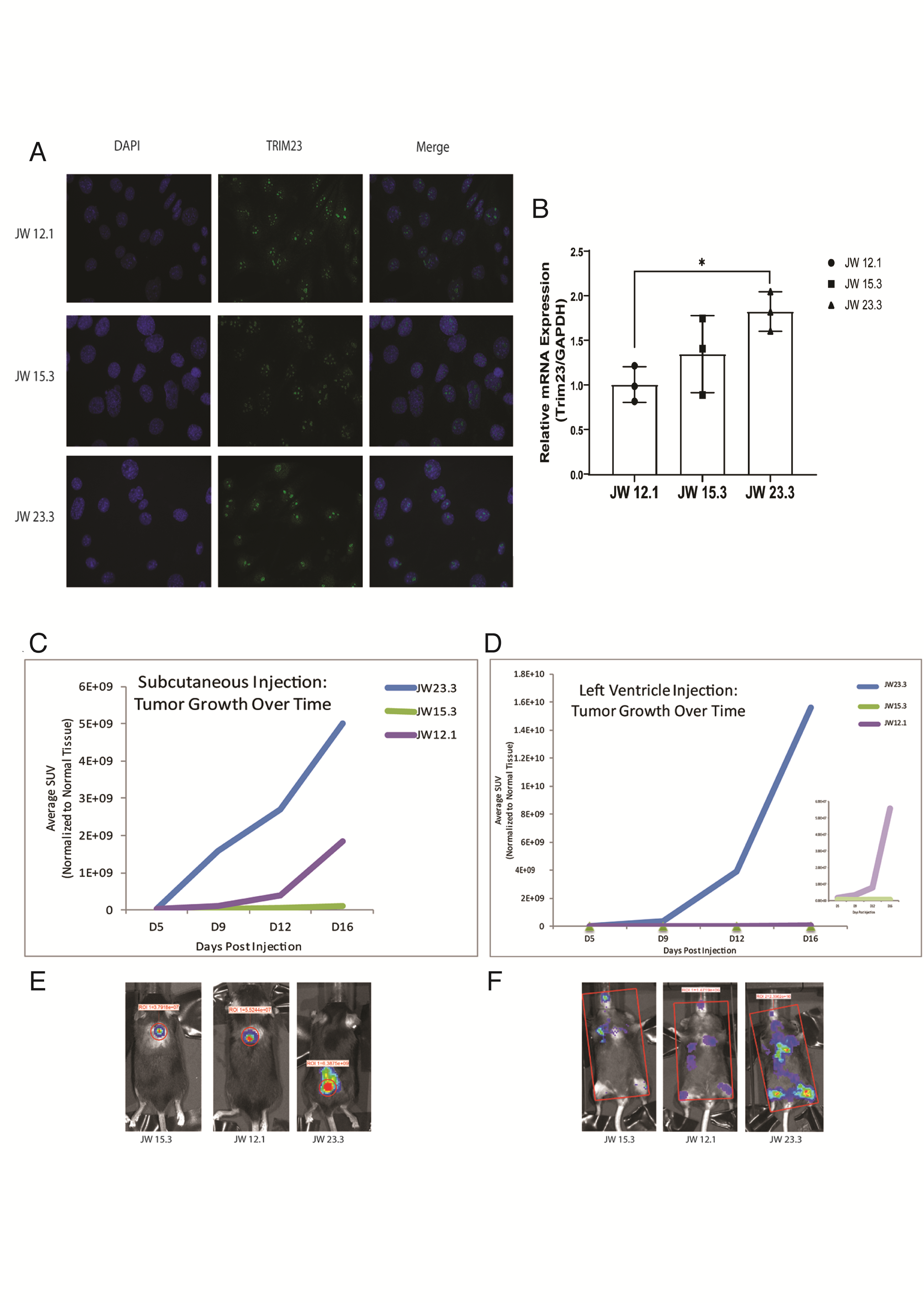

Supplement: vdz026_suppl_Supplementary_Figure_5 [file vdz026_suppl_supplementary_figure_5.png]

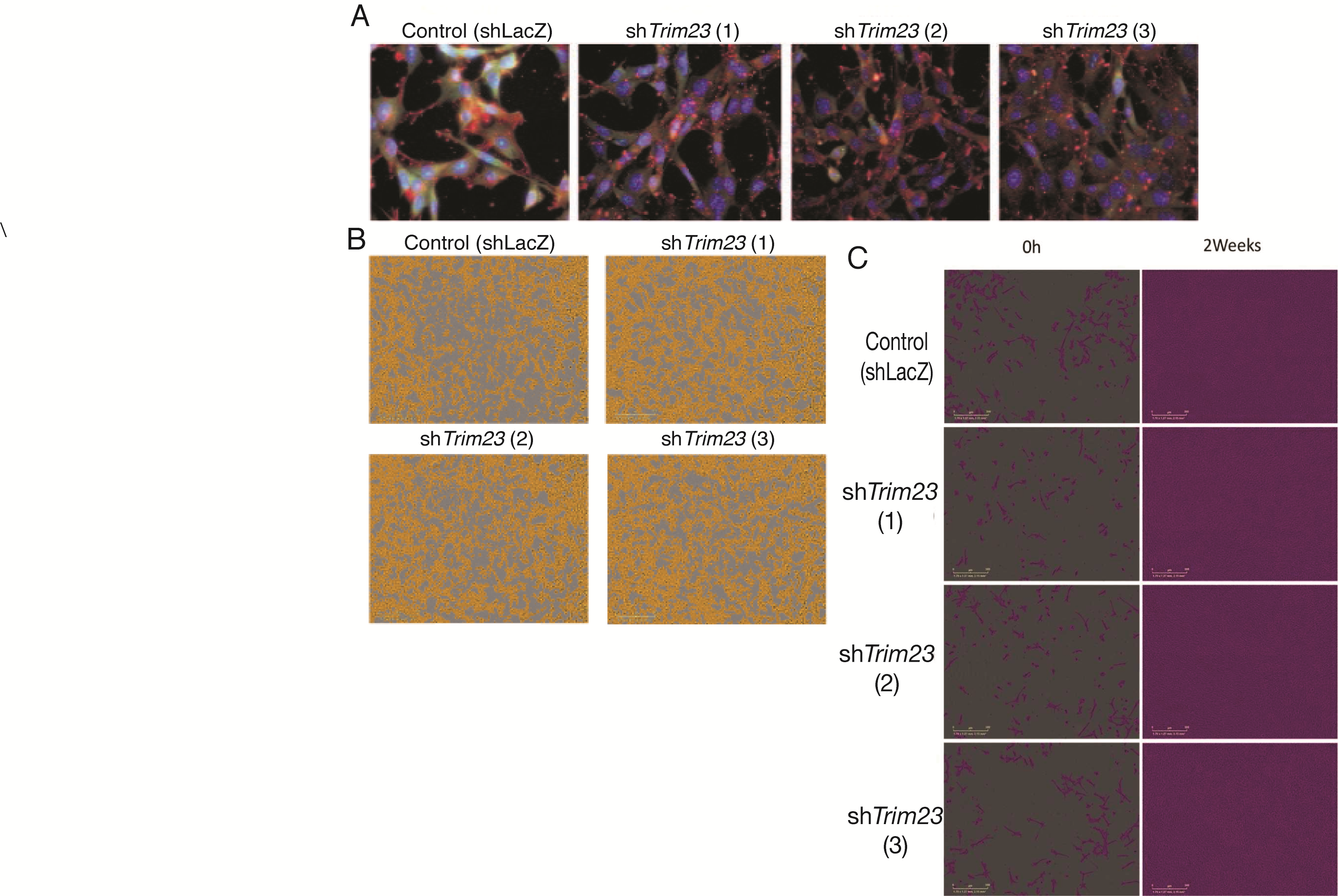

Supplement: vdz026_suppl_Supplementary_Figure_6 [file vdz026_suppl_supplementary_figure_6.png]

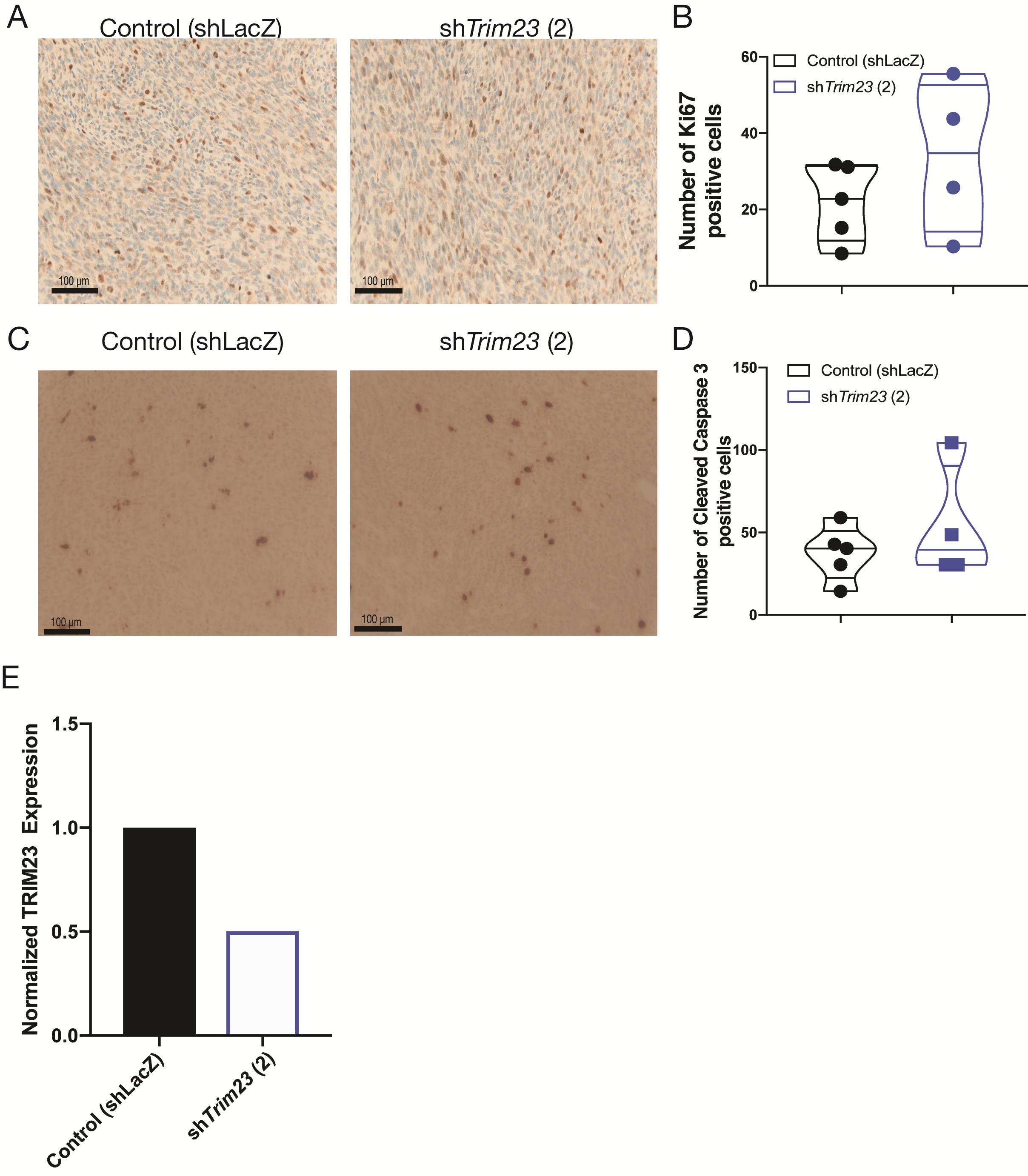

Supplement: vdz026_suppl_Supplementary_Figure_7 [file vdz026_suppl_supplementary_figure_7.png]
