# Supplementary material for: Whole exome sequencing reveals the maintained polyclonal nature from primary to metastatic malignant peripheral nerve sheath tumor in two patients with NF1
Source: Neurooncol Adv. 2019 Sep 10;2(Suppl 1):i75–84. doi: 10.1093/noajnl/vdz026 (PMC7317063; doi:10.1093/noajnl/vdz026)
Supplement: Supplementary file 3 [file vdz026_suppl_supplementary_figure_3.docx]

	Patient A			Patient B		
Gene	Primary	Bone	Lung	Primary	Lung 1	Lung 2
TRIM2		-1.05	-21.48	-1.09	-1.95	-1.01
TRIM3	0.61		-1.58	0.91	0.61	1.05
TRIM4	0.64	1.15	2.47	1.13	0.77	0.78
TRIM5			-2.39	-1.03	0.80	0.81
TRIM6-TRIM34	0.64		-1.32	-1.22	-1.06	1.23
TRIM7	-1.19		-21.76	0.95	1.49	0.97
TRIM8	0.64	-1.07	2.63	-1.29	1.33	0.91
TRIM9	0.66		0.98	0.80	0.68	1.04
TRIM11			0.90			
TRIM14	0.65	-1.03	0.91	-1.01	-1.06	
TRIM16	0.68		-1.64	-1.08		-1.07
TRIM16L	0.64	-1.15	0.73	-1.04	-1.20	
TRIM17		-1.03	-1.36	0.70	1.01	0.77
TRIM21	0.64		1.10	-1.56		0.63
TRIM22		-1.11	1.28			1.32
TRIM23	-1.38	0.99	0.96	0.92	0.98	1.26
TRIM24	-1.18	0.71	-2.64	-1.97	-1.44	0.74
TRIM25	0.70	0.70	1.13	-1.16	0.65	0.74
TRIM27	-1.48	-1.82	-21.23			
TRIM28	0.67		1.15			
TRIM29	0.72	0.68	1.04	-1.38		-1.17
TRIM33	0.74	0.78	1.36	-1.24	-1.63	-1.33
TRIM35	-2.21		-1.38			-1.10
TRIM36	-1.12	0.67	1.42	-1.25	-1.16	1.02
TRIM37	0.84	1.26	1.20	1.26	1.50	0.74
TRIM41	0.75	1.03	0.91	1.29	1.43	1.03
TRIM42			1.12	-1.10	-1.19	
TRIM43	1.25	0.79	0.90	0.77		
TRIM43B	-2.20	-3.42	-20.91	0.66		
TRIM44	0.63		1.31	-1.04	0.64	1.22
TRIM45	0.78		0.99	0.99	0.60	0.62
TRIM46	1.03	0.69	-1.09	0.96	0.97	0.92
TRIM47	0.96	0.87	1.04	0.79	1.08	-1.33
TRIM48			-20.51			
TRIM49		0.89	-1.10			0.62
TRIM49B			0.71		-1.25	
TRIM49C	-1.06	1.03	1.91		-1.16	
TRIM50		0.94	1.63	0.75	0.68	0.84
TRIM51					-1.38	-1.13
TRIM52		0.67	0.82	0.61	1.09	0.74
TRIM54	0.83		1.49	0.61	0.65	0.84
TRIM55	0.98		1.67	0.77	0.61	0.64
TRIM56		0.79			-1.02	0.75
TRIM58	-1.39		-1.10		-1.12	
TRIM59-IFT80	-2.61	-2.25	1.15	1.23	0.90	1.48
TRIM60			-21.36	-1.61	-1.11	-1.08
TRIM61		-1.12			-1.45	
TRIM62	1.34	0.75	1.15	-1.00	-1.24	
TRIM63	0.75		1.28	-1.19	-1.33	-1.46
TRIM64	-1.39	-23.16	-19.82	-3.27	-2.77	0.91
TRIM64B	-1.01	1.01	1.65			0.79
TRIM64C			1.19		-1.58	
TRIM66	0.60		-1.54	0.86	0.97	1.02
TRIM67	-1.83	-2.08	1.74	0.80	0.74	1.11
TRIM68	-1.36		-1.01			0.91
TRIM69			-1.25	-1.03	-1.67	0.86
TRIM71	0.69		0.81			0.71
TRIM72	-1.19	0.71	0.72	-1.86	0.92	-1.05
TRIM73		0.85	-2.05		0.67	0.65
TRIM74	-1.48	1.15	0.99			
TRIM77	-1.02		1.30	-1.25	-1.26	0.92
